# Theory and Applications of Bioinspired Neural Intelligence for Robotics and Control

**DOI:** 10.1155/2016/5089767

**Published:** 2016-08-14

**Authors:** Simon X. Yang, Chaomin Luo, Howard Li, Jianjun Ni, Jianwei Zhang

**Affiliations:** ^1^School of Engineering, University of Guelph, Guelph, ON, Canada N1G 2W1; ^2^University of Detroit Mercy, Detroit, MI 48221, USA; ^3^University of New Brunswick, Fredericton, NB, Canada E3B 5A3; ^4^College of IOT Engineering, Hohai University, Changzhou 213022, China; ^5^University of Hamburg, 22527 Hamburg, Germany

Computational intelligence approaches are nature-inspired methods, which offer a wealth of ideas for solutions to complex problems. In comparison to the traditional approaches, the computational intelligence approaches are more powerful so that they do not need the reformulation of the problem to search a nonlinear and a nondifferentiable space with real world conditions with the massive parallelism. Another advantage of the computational intelligence approaches is the flexibility of the fitness function formulation, which can be expressed as a proper function of the system outputs and are suitable for multiobjective problems.

Recently, a new type of computational intelligence methods has been developed to overcome the limitations of traditional artificial intelligent methods. One of the most important features of these computational intelligence methods is that their working mechanisms are more lifelike to an individual or a group of organisms, which can be understood very well. These methods usually have higher efficiency than the traditional artificial intelligent methods. The computational intelligence methods of this type are defined as bioinspired intelligent algorithms to distinguish from the traditional artificial intelligent methods.

Research on computational intelligence and neuroscience, particularly bioinspired intelligence, has made significant progress in both understanding the neuroscience and biological systems and applying to various robotic and control systems. The aim of this special issue is to provide a forum for different research efforts towards new activities in intelligent robotics and control systems with emphasis on humanoid robots and bioinspired robotics, where the methodologies for the robotic systems are mainly inspired from the strategies, mechanisms, and functionality of neural systems and biological systems, for example, biologically inspired neural networks, genetic algorithms, and fuzzy systems.

This special issue includes six papers selected on a peer review basis. These papers present research results in tracking control of underactuated ship, coordinated path following of multiple marine vessels, driving behaviour modeling of electrical wheelchair users, gait planning and stability control of a quadruped robot, and optimization control of a production process. In addition, there is a survey paper in bioinspired intelligent algorithm and its applications for mobile robot control.

The paper, which is by J. Yuan et al., is about the course control of underactuated surface vessel. In this paper, a backstepping controller with robust neural network is designed to deal with the uncertain and underactuated for the ship. The uniform stability and the convergence of tracking error to zero are quarantined by the Lyapunov stability theory.

The paper by J. Ni et al. presents a survey of the state-of-the-art research in bioinspired intelligent algorithms (BIAs), which focuses on the realization of various BIAs based on different working mechanisms and the applications for mobile robot control, to help in understanding BIAs comprehensively and clearly. This survey paper includes four primary parts: a classification of BIAs from the biomimetic mechanism, a summary of several typical BIAs from different levels, an overview of current applications of BIAs in mobile robot control, and a description of some possible future directions for research.

The paper presented by M. Fu and Y. Xu focuses on the coordinated path following of multiple marine vessels with speed saturation. The objective is to avoid the tracking error jump and solve the speed saturation problem in straight path. The virtual leader strategy is applied, and the neural dynamic model and passivity-based techniques are used together to yield a distributed control strategy.

The paper, which is authored by J. Li et al., is about the gait planning and stability control of the quadruped robot. This paper presents a new CPG (central pattern generator) model controller and its gait switching strategy based on the Wilson-Cowan model, to generate smooth gait and shorten the adjusting time of the model oscillation system. An adaptive speed adjustment and gait switch are completed by the real-time computing of ZMP (zero moment point), to realize stability control.

The paper by S. O. Onyango et al. discusses the driving behaviour modeling problem of electrical wheelchair users. This paper presents the method to obtain the steering data for parameter identification. The modeling method based on the improved Directed Potential Field (DPF) for trajectory planning is applied.

The paper authored by D. He et al. is on the optimization control of the color-coating production process (CCPP). An effective optimization control strategy for the CCPP is proposed. To manage the model uncertainty, the robust optimization approach is introduced to improve the feasibility of the optimized solution and the iterative learning control is then utilized to further refine the model uncertainty.

In order to deal with the upcoming challenges in robotics and control, it is necessary to develop more advanced bioinspired neural intelligence techniques and theories in the literature. This special issue not only captures a snapshot of the current research but also gives some future aspects. We hope it will motivate the readers to direct their effort into this rewarding area.

